# Highly transparent PAA-functionalized porous silica coatings with underwater superoleophobicity and oil-fouling resistance

**DOI:** 10.1039/d6ra05313k

**Published:** 2026-07-13

**Authors:** Lianyi Xu, Jinlong Li, Rongbin Li, Xuemin Lu, Miaosen Yang, Pan Gao, Liping Tong, Su Zhao, Haiyang Jiang

**Affiliations:** a School of Materials Science and Engineering, Shanghai Dianji University Shanghai 201306 P.R. China xuly@sdju.edu.cn; b School of Chemistry and Chemical Engineering, State Key Laboratory of Metal Matrix Composite, Shanghai Jiao Tong University 800 Dongchuan Road Shanghai 200240 China

## Abstract

Transparent underwater superoleophobic coatings hold great promise for underwater optics and marine devices, but their practical application is hindered by the inherent conflict between optical transparency, mechanical robustness, and oil-repellent durability. Here, we report a high-performance transparent underwater superoleophobic (TUS) coating designed by covalently anchoring high-molecular-weight poly(acrylic acid) chains onto a robust, porous nano-silica scaffold. The highly hygroscopic PAA chains form a stable hydration layer on the nanostructured surface, which is critical for achieving underwater oil repellency. This rational design combines mechanical stability with a stable hydration layer, enabling the coating to exhibit high transparency (92.3% in water at 550 nm), robust underwater superoleophobicity (oil contact angle: 169° ± 2°; sliding angle: 1.5° ± 0.3°), and near-zero oil adhesion (adhesion force ≈ 0.48 µN). Crucially, the TUS coating demonstrates good durability under the tested conditions, maintaining its anti-oil-fouling performance and optical clarity after prolonged exposure to high-salinity environments (20 days in 3.5 wt% NaCl), elevated temperatures (up to 70 °C), hydrodynamic shear (0.5 m s^−1^ for 12 hours), and mechanical abrasion. This work provides a feasible strategy for designing multi-functional coatings for demanding applications in underwater optical and marine equipment.

## Abstract 2

A transparent, robust, and durable underwater superoleophobic coating yields from a PAA-grafted porous silica design.

## Graphical abstract

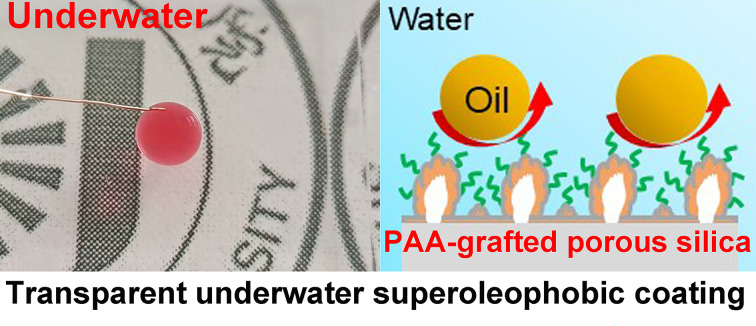

## 1 Introduction

Transparent underwater superoleophobic coatings are of significant scientific and industrial interest for their potential applications in underwater optics, marine engineering, and biomedical devices.^1–4^ These coatings combine exceptional resistance to oil fouling in aquatic environments with high optical transparency.^4–6^ Inspired by natural prototypes such as fish scales, which exhibit remarkable oil-repellency underwater due to their hydrophilic micro-scaled structures, researchers have developed various artificial underwater superoleophobic surfaces.^7–9^ These surfaces are typically constructed from hydrophilic materials, including hydrogels,^10–12^ metal oxides,^13^ and hydrophilic polymers,^7^ and are fabricated *via* techniques such as biomineralization,^2,14,15^ etching,^16^ templating,^17^ electrochemical deposition,^18^ spray coating,^19^ and self-assembly.^20,21^ The fundamental principle behind underwater superoleophobic surfaces lies in their ability to trap water molecules within hierarchical micro/nanostructures, forming a hydrated layer that prevents oil adhesion.^2,3^ This phenomenon, known as the “Cassie–Baxter” state, creates an energy barrier that repels oil droplets, resulting in underwater oil contact angles (UWOCA) greater than 150°.^7,12^ However, a major challenge is that the surface roughness necessary for underwater superoleophobicity often scatters visible light, compromising optical transparency.^22–24^ The limitation severely restricts the use of such coatings in optical devices such as diving goggles, underwater cameras, and solar panels, where high transparency is essential.^2,25,26^

Substantial research has been dedicated to resolving the inherent conflict between oil-repellency and optical transparency in underwater superoleophobic surfaces.^1,27,28^ For instance, Teng *et al.* developed a transparent nanocomposite hydrogel with enhanced mechanical strength by incorporating clay and using a filtration method to create a rough surface and an interior layered structure.^12^ Gu *et al.* fabricated a robust coating through an ultra-facile immersion-curing process, though its transparency was compromised by the composite nature of the material.^27^ Li *et al.* also produced a robust amorphous calcium phosphate (ACP) coating *via* a facile mineralization strategy, which exhibits excellent anti-fogging and anti-crude-oil-fouling performance.^4^ Despite these advancements, most existing studies have focused on achieving static superoleophobicity under mild conditions, while the long-term durability of these coatings under realistic operational environments, such as high salinity, elevated temperatures, hydrodynamic shear, and mechanical abrasion, remain largely unaddressed.^2,3^ In practical applications, these coatings are subjected to such abrasive and shear forces, which can readily degrade the delicate hydrophilic layer.^2^ Conventional reinforcement strategies, such as increasing the crosslink density,^24^ incorporating inorganic nanoparticles,^22^ or adding micro-fillers,^29^ often inadvertently introduce light-scattering centers that diminish transparency. Furthermore, ensuring robust interfacial adhesion under prolonged immersion is particularly difficult. Many coatings suffer from interfacial failure triggered by water-induced swelling, differential thermal expansion, or chemical degradation at the coating-substrate interface.^2,3^ This issue is especially acute for transparent coatings, where traditional opaque primers or adhesion promoters cannot be used.^5^ Consequently, there is an urgent need for innovative coating architectures that not only achieve high transparency and underwater superoleophobicity but also maintain these properties under prolonged exposure to harsh environmental conditions.

Porous nano-silica (SiO_2_) has emerged as a particularly promising platform for constructing underwater superoleophobic coatings, owing to its tunable size, controllable morphology, and rich surface chemistry.^7^ The precise nanoscale engineering of silica structures enables the creation of surfaces that concurrently satisfy the roughness criteria for superoleophobicity and the dimensional constraints for optical transparency, by keeping structural features below the wavelength of visible light to minimize scattering.^30,31^ Building upon this foundation, our previous work developed highly transparent and mechanically robust superhydrophobic surfaces from an electrodeposited porous poly(3,4-ethylenedioxythiophene) (PEDOT) film encased in silica.^32^ Subsequent calcination yielded a hollow porous silica coating that retained high transparency, mechanical stability and exhibited superhydrophilicity, thereby providing an ideal scaffold for subsequent functionalization. We therefore hypothesized that grafting hydrophilic polymers onto this robust, nanostructured silica scaffold could yield a coating that synergistically combines the mechanical stability of the inorganic framework with the hydration capability of the polymer, potentially addressing the longstanding challenge of durability in transparent underwater superoleophobic coatings.

In this work, we report a transparent underwater superoleophobic (TUS) coating that demonstrates good tolerance to a broad spectrum of physical and environmental challenges. Polyacrylic acid (PAA) was selected because of its high hygroscopicity, thermal stability, optical transparency in the visible range, and abundant carboxyl groups for covalent anchoring. Nano-silica provides a mechanically robust, optically transparent, and easily functionalizable porous scaffold. Their combination reconciles the conflicting requirements of transparency, mechanical stability, and underwater superoleophobicity.^7,28,33,34^ The coating was fabricated by grafting PAA (*M*_v_ ≈ 1 250 000) onto the porous nano-silica scaffold prepared on indium tin oxide (ITO) glass *via* our established electrochemical templating method. The PAA chains effectively modify the porous nanostructure, ensuring the formation of a stable hydration layer atop the scaffold. The synergistic combination of the mechanically stable silica scaffold and the highly hygroscopic PAA endows the TUS coating with both high optical transparency (92.3% transmittance in water and 82.9% in air at 550 nm) and robust underwater superoleophobicity (underwater oil contact angle, UWOCA = 169° ± 2°; underwater oil sliding angle, SA = 1.5° ± 0.3°). Notably, the TUS coating maintains its anti-oil-fouling performance even after exposure to high salinity, elevated temperatures, hydrodynamic shear, and mechanical abrasion, thereby demonstrating its potential for demanding real-world applications in underwater optical and marine environments.

## 2 Experimental

### 2.1 Materials

HPLC grade acetonitrile (ACN) was provided by Shanghai Lingfeng Chemical Reagent Company. 3,4-Ethylenedioxythiophene (EDOT, 99%), (3-aminopropyl)triethoxysilane (APTES, 98%), *n*-hexadecane (98%), tetraethoxysilane (99%), and diiodomethane (CH_2_I_2_, 99%) were purchased from Adamas and used as received. Poly(acrylic acid) (PAA, average *M*_v_ ≈ 1 250 000) was obtained from Aldrich and used without further purification. Anhydrous lithium perchlorate (LiClO_4_, 99%) was obtained from J&K and used as received. 1,2-Dichloroethane (DCE, 99%), dimethylformamide (DMF, 99.8%), toluene (99.5%), acetone (99.5%), and absolute ethanol (99.5%) were purchased from Greagent and used as received. Mineral oil (150 SN) and peanut oil (food grade) were sourced from a local supermarket. Waste engine oil was procured from a local market.

### 2.2 Fabrication of TUS coatings *via* PAA grafting

Highly transparent and mechanically stable porous nano-silica (TMNS) coatings were fabricated on indium tin oxide (ITO) glass substrates following our previous report (*J. Mater. Chem. A*, 2015, **3**, 3801–3807). Briefly, conductive PEDOT templates were first electrodeposited onto the ITO, followed by a 48 hours chemical vapor deposition (CVD) of tetraethoxysilane (TEOS) precursors (for details, see Section S1, SI). A subsequent controlled calcination at 530 °C for 2 hours removed the organic templates and generated a porous nanostructure. To impart underwater superoleophobicity, the TMNS coatings were functionalized with poly(acrylic acid) (PAA) *via* a two-step process. First, aminosilanization was performed by immersing the coatings in a mixture of absolute ethanol (200 mL) and deionized (DI) water (20 mL) containing 500 µL of (3-aminopropyl)triethoxysilane (APTES), with stirring at 250 rpm for 24 h at room temperature. After being rinsed with ethanol and dried under ambient conditions, the aminosilanized samples were then immersed in a solution of PAA (*M*_v_ ≈ 1 250 000, 0.2 g) in *N*,*N*-dimethylformamide (DMF, 400 mL). The grafting reaction was conducted under a nitrogen atmosphere at 130 °C for 24 h with stirring in a reflux system. Finally, the resulting functionalized coatings, denoted as TUS coatings, were thoroughly rinsed with DI water and dried at room temperature prior to characterization. The PAA chains are covalently attached *via* amide bonds formed between –COOH and –NH_2_, leading to a multipoint-anchored conformation rather than a classical polymer brush configuration.

### 2.3 Contact angle measurements

Static contact angles (CAs) in air were measured using the sessile drop method with a Contact Angle System OCA 20. The reported values are the mean of three independent measurements, performed with 4 µL water droplets or 6 µL droplets of organic liquids. For underwater superoleophobicity evaluation, the samples were first immersed in deionized water to achieve complete pre-wetting. The UWOCAs were then measured with the same instrument. The corresponding SAs were determined by tilting the stage at a rate of 0.5°·s^−1^ and recording the angle at which a 6 µL oil droplet (dichloroethane) began to move. For oils less dense than water (*e.g.*, hexane), the captive bubble method was employed to measure the contact angles.

### 2.4 Characterization

The surface morphology was characterized by field-emission scanning electron microscopy (FE-SEM; Nova NanoSEM, FEI). Chemical composition was analyzed by X-ray photoelectron spectroscopy (XPS) on a Kratos Axis UltraDLD spectrometer using monochromatic Al-Kα radiation (1486.6 eV) at a 90° takeoff angle. Fourier-transform infrared (FTIR) spectra were recorded on a Bruker VERTEX 70 spectrometer using KBr pellets. The surface topography and roughness were quantified by atomic force microscopy (AFM; Bruker Dimension Icon) operating in tapping mode. The root-mean-square roughness (*R*_q_) was derived from five randomly selected 1 × 1 µm^2^ areas. Optical transmittance spectra (380–950 nm) were acquired on a Shimadzu UV-2600i UV-vis spectrophotometer operating in double-beam mode. The adhesion force between underwater oil droplets and the TUS coating was measured using a DataPhysics DCAT11 microelectromechanical balance system. In this test, a 6 µL dichloroethane droplet suspended from a metal ring was approached to and retracted from the sample surface at 0.01 mm s^−1^ in water, with the maximum retraction force in the force–distance curve defined as the adhesion force. The refractive index and thickness of the coating were determined by spectroscopic ellipsometry (J. A. Woollam AutoRetarder™ W-VASE). The anti-fogging performance was assessed by exposing samples to 60 °C steam for 10 min, with surface clarity documented using a digital camera under controlled lighting. Thermal stability was determined by measuring the UWOCA and SA over a 25–70 °C range in a thermostatic bath. Mechanical durability against hydrodynamic shear was evaluated by exposing the coating to a 0.5 m s^−1^ water stream for 12 h.

### 2.5 Particle impact abrasion test

The mechanical abrasion resistance was evaluated using a particle impact test. Titanium powder (quasi-spherical, 5–80 µm) was used as the model abrasive. The sample was fixed at a 45° tilt angle in air. Particles were dropped from a height of 15 cm (impact velocity ≈ 1.7 m s^−1^) at a controlled flux of 0.1 g s^−1^ for 10 s, concentrated onto a fixed circular spot (*Φ* ≈ 10 mm) on the coating surface. After the impact test, the sample was gently cleaned with air. The surface morphology was examined by SEM, and the UWOCA (using DCE) was measured on the impacted area.

## 3 Results and discussion

### 3.1 Fabrication of TUS coatings

**Fig. 1:**
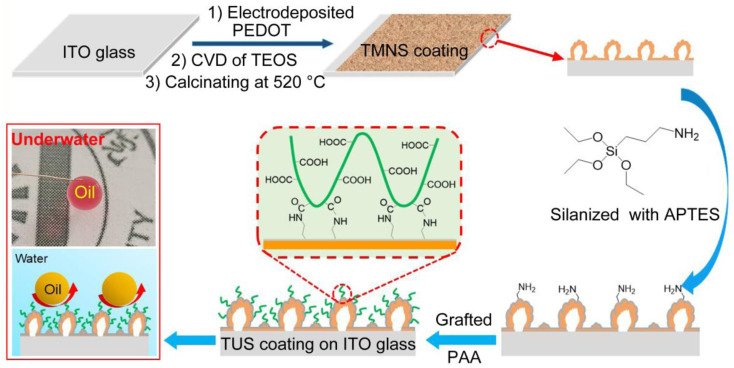
Schematic illustration of the TUS coating fabrication process on ITO glass. Inset: digital photograph demonstrating the coating's transparency and underwater superoleophobicity.

The TUS coating was constructed through a rational design that grafts poly(acrylic acid) (PAA) onto a highly transparent and mechanically robust porous nano-silica (TMNS) scaffold (Fig. 1). This design combines a robust inorganic scaffold with a functional organic polyelectrolyte. The TMNS underlayer itself was synthesized *via* an established electrochemical templating strategy (Fig. S1), wherein a calcination step at 530 °C not only removed the organic template to generate a porous nanostructure but also formed robust covalent Si–O–metal bonds at the silica-ITO interface. This process yielded a TMNS coating exhibiting >92% optical transmittance and exceptional mechanical adhesion (>15 MPa by scratch testing), providing an ideal substrate. Subsequent functionalization was achieved by first introducing amine groups *via* APTES silanization, followed by covalent grafting of high-molecular-weight PAA (*M*_v_ ≈ 1 250 000) through thermal amidation. The porous nanostructure of the TMNS scaffold offers a high surface area for dense PAA grafting, while the inherent hygroscopicity of PAA ensures the formation of a stable interfacial hydration layer. The synergistic combination is critical for achieving the exceptional and stable underwater superoleophobicity observed in the final TUS coating.

### 3.2 Morphology and chemical composition

**Fig. 2:**
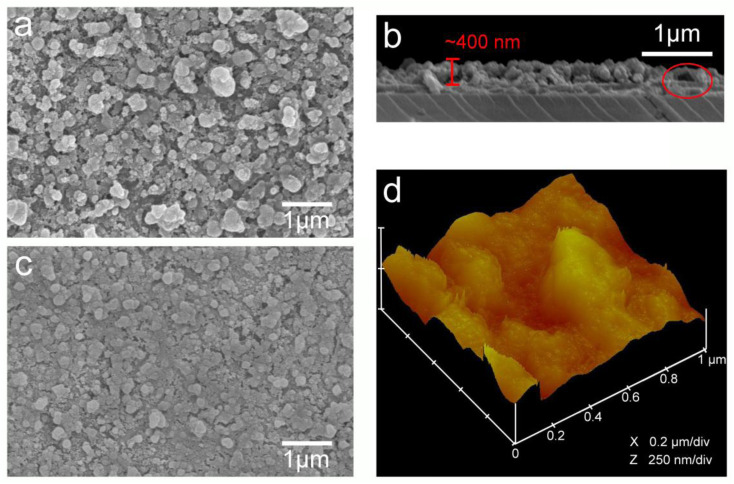
Morphology and topography of the TMNS and TUS coatings. (a) Plan-view SEM image of the TMNS coating (300 00×), revealing a hierarchical porous nanostructure derived from sacrificial PEDOT templates. (b) Cross-sectional SEM image of the TMNS coating (413 10×), showing a uniform coating thickness of approximately 400 nm. (c) SEM image of the TUS coating after PAA functionalization (300 00×), confirming the retention of the porous structure essential for underwater superoleophobicity. (d) AFM topography image of the TUS coating (scan size = 1 µm × 1 µm, *z*-scale = 250 nm), demonstrating a preserved nanoprotrusive surface morphology with a root-mean-square roughness (*R*_q_) of 38.5 ± 5.2 nm.

Scanning electron microscopy (SEM) revealed that the TMNS coating on ITO glass possesses a hierarchical porous nanostructure (pore size: 20–400 nm) with a uniform thickness of approximately 400 nm (Fig. 2a). Cross-sectional SEM imaging (Fig. 2b) confirms that this architecture originates from sacrificial PEDOT templates, evident from the hollow silica frameworks that replicate the templated cavities (Fig. S2). The thin-walled structure (wall thickness ∼100 nm) contributes to high optical transparency by minimizing light scattering, achieved through refractive index matching between the silica matrix and air-filled pores. Following PAA functionalization, the resulting TUS coating retains this hierarchical porosity (Fig. 2c), which is essential for stabilizing the interfacial water layer responsible for its underwater superoleophobicity. Atomic force microscopy (AFM) further verified the preservation of nanoprotrusive topography after PAA grafting (Fig. 2d), with a root-mean-square roughness (*R*_q_) of 38.5 ± 5.2 nm (averaged over five 1 × 1 µm areas) (Fig. 2d and S3). Although the PAA layer induces subtle morphological changes, the organic–inorganic hybrid architecture enhances the underwater superoleophobic performance by synergistically promoting capillary-driven water trapping within the hierarchical nanostructures and stabilizing the hydration layer through hygroscopic polymer–water interactions.

**Fig. 3:**
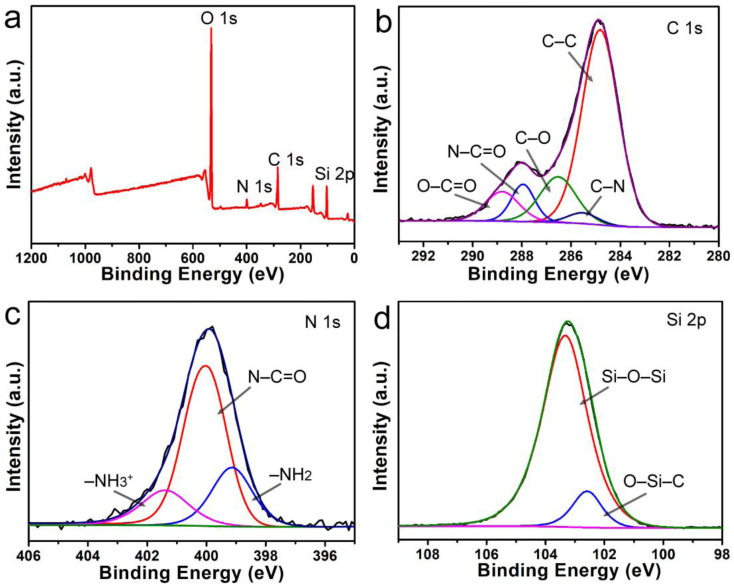
(a) XPS survey spectra of the TUS coating. High-resolution XPS spectra of (b) C 1s, (c) N 1s, and (d) Si 2p core levels for the TUS coating.

X-ray photoelectron spectroscopy (XPS) and Fourier transform infrared (FTIR) spectroscopy provide conclusive evidence for the covalent functionalization of the TUS coating. The XPS survey spectrum (Fig. 3a) confirms the introduction of diagnostic elements, showing emergent nitrogen (from APTES silane) and a substantially enhanced carbon signal, which collectively indicate successful PAA grafting. The high-resolution C 1s spectrum (Fig. 3b) resolves five distinct chemical states at 284.8 eV (C–C/C–H), 285.6 eV (C–N), 286.5 eV (C–O), 287.9 eV (amide N–C=O), and 288.8 eV (carboxylate O–C=O). The coexistence of amide and carboxylate peaks unequivocally confirms the covalent grafting of PAA through amide bond formation. This conclusion is further validated by the N 1s spectrum (Fig. 3c), wherein the characteristic amide peak at 400.0 eV (N–C=O) is observed alongside residual APTES signatures-amine (–NH_2_, 399.1 eV) and protonated ammonium (–NH_3_^+^, 401.4 eV). Concurrently, the Si 2p spectrum (Fig. 3d) demonstrates stable APTES chemisorption *via* a distinct O–Si–C siloxane bridge signal at 102.6 eV, adjacent to the substrate SiO_2_ peak at 103.3 eV. FTIR analysis (Fig. S4) reveals conclusive evidence for amide bond formation, manifested by a redshifted carbonyl stretch from 1711 cm^−1^ to 1664 cm^−1^ (amide I band) and the emergence of a characteristic amide II band at 1554 cm^−1^^7^ These spectral shifts confirm the conversion of carboxylic groups to amide linkages. The integrated spectroscopic data confirm the successful surface functionalization *via* amide covalent bonds and siloxane networks, establishing a chemically stable platform for underwater superoleophobicity.

### 3.3 Superhydrophilicity and underwater superoleophobicity

**Fig. 4:**
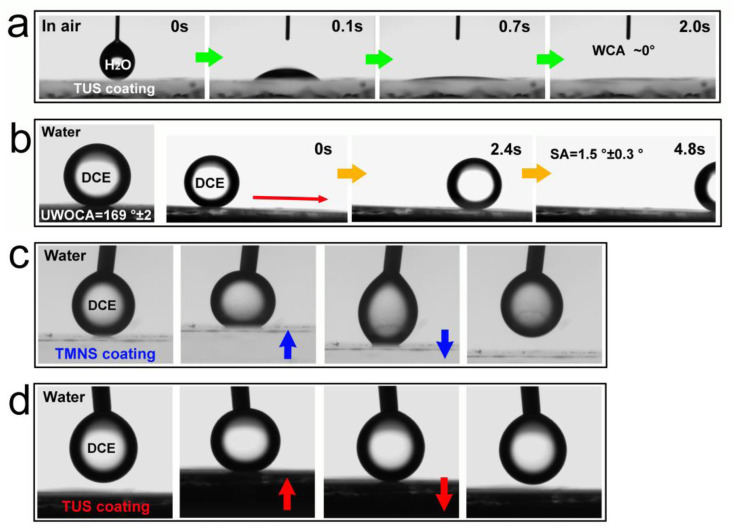
Wettability characterization of the TUS coatings. (a) A water droplet (4 µL) spontaneously spreading on the TUS coating in air, indicating superhydrophilicity (WCA ≈ 0°). (b) An underwater oil droplet (DCE, 6 µL) on the TUS coating, showing a high UWOCA of 169° ± 2° and a low SA of 1.5° ± 0.3°, demonstrating its underwater superoleophobicity and low adhesion. (c and d) Adhesion test *via* cyclic compression-detachment of a DCE droplet (6 µL) underwater on (c) the TMNS coating and (d) the TUS coating, revealing strong adhesion and complete repellency, respectively.

The wettability of TUS coatings (electrodeposited at charge densities of 32.6 mC cm^−2^) was characterized in air and underwater. In air, a 4 µL water droplet spontaneously spreads and completely wets the surface within 2.0 s, achieving a water contact angle (WCA) of ∼0° (Fig. 4a and Movie S1). This rapid wetting kinetics confirms superhydrophilicity, attributed to the capillary effect induced by its hydrophilic porous nanostructure. Remarkably, a distinct wettability transition occurred underwater. A 6 µL dichloroethane (DCE, *ρ* = 1.25 g mL^−1^) droplet deposited onto TUS coating surface underwater exhibits a static UWOCA of 169° ± 2° (Fig. 4b), confirming its underwater superoleophobicity. The TUS coating demonstrates an exceptionally low SA of 1.5° ± 0.3° with rapid roll-off (<5.0 s), evidencing ultralow adhesion and anti-fouling properties (Fig. 4b, right, and Movie S2). After cyclic compression-detachment tests of DCE droplets on submerged TUS coatings, zero residual oil was observed (Fig. 4d and Movie S3). Quantitative assessment revealed extremely low oil contact angle hysteresis (Δ*H*_oil_ = *θ*_a_ − *θ*_r_ = 2° ± 1°; *θ*_a_ = 170.5° ± 1°; *θ*_r_ = 168.5° ± 1°), confirming a stable Cassie state (Movie S4).^3^ In contrast, while the TMNS coating also exhibited a high UWOCA for DCE (∼150°), it demonstrated strong oil adhesion under the same compression-detachment tests, indicating an unstable, adhesive state (Fig. 4c and Movie S5). The robust underwater superoleophobicity of TUS coating originate from high-molecular-weight PAA chains covalently grafted onto the porous nano-silica scaffold. The hydrophilic PAA layers form a densely hydrated barrier through hydrogen bonding with water molecules and trap water within their porous nanostructure. This creates a thermodynamically stable solid–water interface that minimizes solid–oil contact, thereby sustaining the Cassie state (Fig. 1).^3,15^

**Fig. 5:**
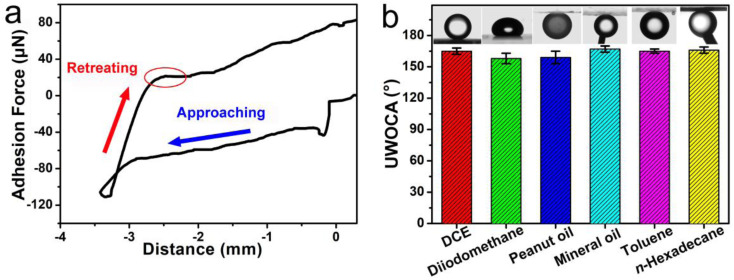
(a) Force–distance curve from a microelectromechanical balance test, revealing an ultralow adhesion force (*F*_ad_ ≈ 0.48 µN) between a DCE oil droplet and the submerged TUS coating. (b) The UWOCAs for oils of varying viscosities on the TUS coating, all exceeding 150°, demonstrating its versatile underwater superoleophobicity.

The interfacial adhesion force between a dichloroethane (DCE) oil droplet and the submerged TUS coating was quantified using a microelectromechanical balance system (Fig. 5a). A 6 µL DCE droplet, suspended from a metal ring in water, served as the testing probe. The coating underwent a controlled approach–contact–retraction cycle at 0.01 mm s^−1^, with synchronous recording of the force–distance profile. An ultralow adhesion force (*F*_ad_ ≈ 0.48 µN) measured during the retraction phase confirms near-zero oil adhesion. The coating's underwater superoleophobicity was further demonstrated against oils of varying viscosity, including toluene (*η* = 0.59 mPa s; UWOCA = 165° ± 2°), diiodomethane (*η* = 2.80 mPa s; UWOCA = 158° ± 5°), *n*-hexadecane (*η* = 3.34 mPa s; UWOCA = 166° ± 3°), peanut oil (*η* ≈ 70 mPa s; UWOCA = 159° ± 6°), and mineral oil (*η* ≈ 135 mPa s at 40 °C; UWOCA = 167° ± 3°). All oils exhibited UWOCAs exceeding 150° (Fig. 5b), demonstrating effective underwater superoleophobicity across a wide viscosity range.

The wetting properties of TMNS coatings and their polyelectrolyte-functionalized counterparts (TUS coatings), synthesized across a series of electrodeposition charge densities (1#: 11.0, 2#: 23.4, 3#: 36.1, 4#: 54.5 mC cm^−2^), are summarized in Table 1. All TMNS coatings were superhydrophilic (WCA ≈ 0°) and exhibited underwater oleophobicity with high adhesion (UWOCA: 141°–151°). In contrast, the TUS coatings achieved persistent underwater superoleophobicity, evidenced by high UWOCA (160°–169°) and ultralow sliding angles (SA: 1.0°–2.0°) over the broad charge density range of 11–54 mC cm^−2^, demonstrating their exceptional interfacial stability and operational consistency. The markedly superior oil-repellent performance of the TUS coatings, relative to both the flat glass baseline (WCA = 34° ± 6°, UWOCA = 133° ± 5°) and the TMNS coatings, is attributed to a synergistic interplay between the porous nanostructures and the grafted PAA functionalization (Fig. S5). Consequently, these findings establish that the concerted combination of hierarchical nanoscale roughness with hydrophilic polyelectrolyte functionalization is a fundamental requisite for achieving persistent, adhesion-free underwater superoleophobicity.

**Table 1:** Comparison of wetting properties between TMNS and TUS coatings fabricated at different electrodeposition charge densities

Sample	WCA (air)	UWOCA (DCE, underwater)	SA (underwater)
Flat glass	34° ± 6°	133° ± 5°	Adhesion
TMNS-1#	∼0°	141° ± 3°	Adhesion
TMNS-2#	∼0°	145° ± 4°	Adhesion
TMNS-3#	∼0°	149° ± 3°	Adhesion
TMNS-4#	∼0°	151° ± 3°	Adhesion
TUS-1#	∼0°	160° ± 3°	2° ± 0.5°
TUS-2#	∼0°	165° ± 2°	1.5° ± 0.3°
TUS-3#	∼0°	168° ± 2°	1.6° ± 0.3°
TUS-4#	∼0°	169° ± 2°	1.0° ± 0.3°

### 3.4 Underwater transparency

**Fig. 6:**
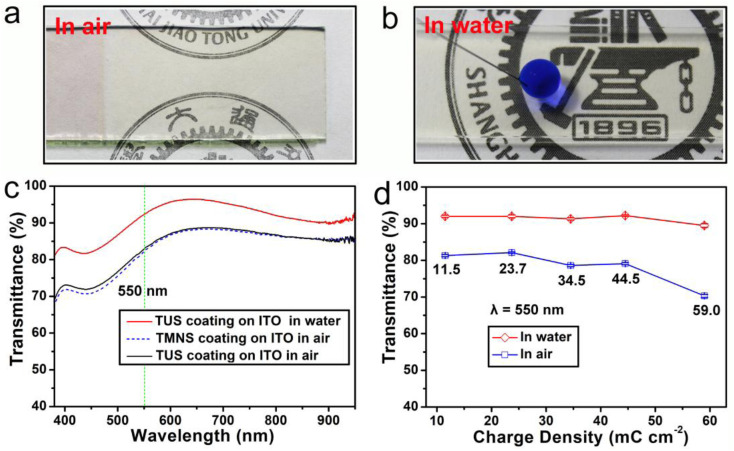
(a and b) Digital photographs of the TUS coating placed over a patterned substrate in (a) air and (b) water, demonstrating high optical clarity in both environments. Spherical, dyed DCE oil droplets in (b) confirm robust underwater superoleophobicity. (c) Optical transmittance spectra of TUS coating in air and water, showing a significant enhancement upon immersion. (d) Transmittance (at 550 nm) of the TUS coatings in both air and water as a function of the PEDOT template's electrodeposition charge density (11.5–59.0 mC cm^−2^).

Underwater superoleophobic surfaces often rely on hierarchical micro/nanostructures that trap water but cause significant light scattering, compromising optical transparency.^2^ The TUS coating overcomes this trade-off by employing a nanostructure with feature sizes smaller than the wavelength of visible light. Specifically, the porous silica scaffold possesses pore diameters in the range of 20–400 nm and wall thickness of approximately 100 nm, both dimensions are below or comparable to the wavelengths of visible light (380–740 nm). Consequently, Mie scattering, which dominates when scattering centers are comparable to the light wavelength, is greatly suppressed in air. As shown in Fig. 6a, the underlying patterned substrate remains clearly visible through the TUS coating. This optical clarity is well preserved upon water immersion (Fig. 6b), confirming excellent underwater transparency. Moreover, when submerged, dyed oil droplets (20 µL DCE, stained with Oil Blue OB) maintain a nearly spherical shape on the coating surface, demonstrating robust underwater superoleophobicity without sacrificing optical clarity.

Optical transmittance measurements confirm the exceptional optical properties of the TUS coating, particularly its significant transmittance enhancement in aqueous environments. The TUS coating, like its underlying TMNS coating (electrodeposited at a charge density of 26.8 mC cm^−2^), exhibits high in-air transparency across the visible spectrum, with transmittance values of 82.9% and 82.2% at 550 nm, respectively (Fig. 6c). Although its in-air transmittance at 550 nm (82.9%) is slightly lower than that of annealed ITO glass (84.4%), the TUS coating exhibits a similar optical response to hydration. When submerged in water, its transmittance increases to 92.3%, corresponding to an absolute gain of 9.4%. Under identical conditions, bare ITO glass achieves a higher final transmittance of 93.8% in water (a gain of 9.4%, Fig. S6). The parallel enhancement trend suggests that the reduction of refractive index mismatch at the interface is a key mechanism in both cases.

The exceptional underwater transparency enhancement originates from the simultaneous reduction of interfacial Fresnel reflections and suppression of Mie scattering within the coating.^15,16^ In air, the coating exhibits high transparency due to its porous nanostructure, which minimizes intrinsic light scattering losses.^2^ Upon water immersion, the nanoscale pores undergo complete infiltration, replacing solid–air interfaces with solid–water interfaces. The infiltration reduces the refractive index contrast between the scattering features (pores per walls) and the surrounding medium, thereby further suppressing any residual Mie scattering. The complete hydration of the silica and PAA components fundamentally transforms the coating's optical properties. Spectroscopic ellipsometry measurements confirm that upon hydration, the effective refractive index of the coating is *n*_eff_ = 1.36, a value remarkably close to that of water (*n* = 1.33). The resulting minimal refractive index contrast (Δ*n* ≈ 0.03) at the coating-water interface not only suppresses Mie scattering but also reduces Fresnel reflection at the coating-water boundary, calculated from the Fresnel equation *R* = [(*n*_1_ − *n*_2_)/(*n*_1_ + *n*_2_)]^2^.^15,16^ Consequently, the TUS coating exhibits exceptionally high transparency in aqueous environments.

Fig. 6d depicts the transmittance of TUS coatings fabricated with varying PEDOT electrodeposition charge densities (11.5–59 mC cm^−2^), measured in both air and water (Fig. S7). In air, transmittance decreases with increasing charge density, directly attributed to enhanced light scattering from the associated increase in surface roughness. In water, however, all samples achieve consistently high transmittance (exceeding 89.5% at 550 nm), with minimal variation across the different charge densities. This result confirms that water penetration effectively counteracts the detrimental optical effects of surface roughness by minimizing refractive index contrasts, thereby ensuring robust and high underwater transparency across all samples.

### 3.5 Durable underwater transparency and oil-fouling resistance

**Fig. 7:**
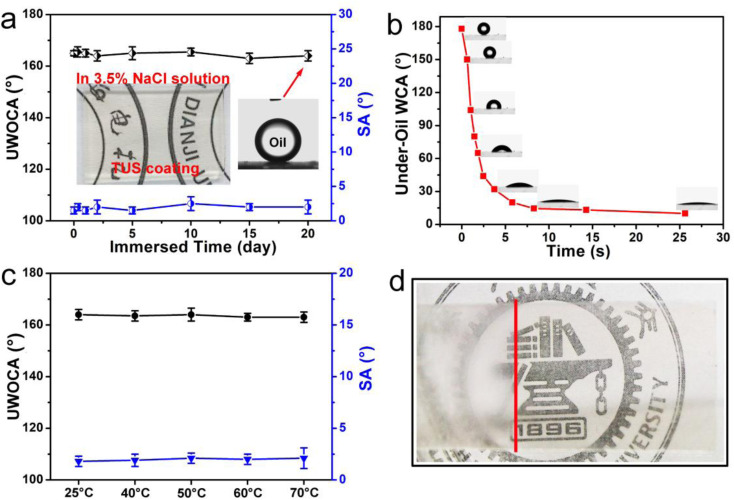
(a) Long-term stability in simulated seawater (3.5 wt% NaCl) over 20 days, maintaining underwater superoleophobicity (UWOCA >150°, SA <5°). Inset: optical photographs of the transparent TUS-coated substrate (left) and the corresponding UWOCA measurement (right) after the immersion test. (b) Dynamic wetting behavior in an under-oil water-wetting test, showing rapid water infiltration (0° in 26 s). (c) Thermal stability across 25–70 °C, with consistent superoleophobicity (UWOCA >150°, SA <5°) demonstrating robust performance at elevated temperatures. (d) Anti-fogging performance in a high-humidity environment, where the coated area (right) retains clarity while the uncoated area (left) is obscured.

The long-term stability of the TUS coating's underwater superoleophobicity was evaluated in a high-salinity environment. The coating was immersed in simulated seawater (3.5 wt% NaCl) for 20 days, and its performance was monitored throughout the period. The coating consistently retained a high underwater oil contact angle (UWOCA >150°) and an ultralow sliding angle (SA <5°), demonstrating exceptional resistance to ionic strength variations over an extended period (Fig. 7a). Simultaneously, it preserved high optical transparency, as evidenced by the clear underlying pattern in the inset of Fig. 7a. The combination of durable saline resistance and optical clarity makes it a highly suitable anti-fouling coating for underwater optical devices.

The underlying wetting mechanism of the TUS coating was further elucidated through an under-oil water-wetting test (Fig. 7b). Upon introducing a water droplet onto the surface submerged in hexadecane, it was rapidly absorbed. The corresponding under-oil WCA exhibited a sharp decay, plunging most significantly within the initial 5 seconds and approaching 0° within 26 seconds (Movie S6). The kinetic profile signifies a highly hydrophilic and porous material. Capillary forces rapidly draw the water droplet into the hydrophilic matrix, displacing the oil and establishing a stable hydration layer. The *in situ*-generated aqueous layer acts as the primary barrier against oil, providing the fundamental mechanism for the observed durable superoleophobicity.

The thermal stability of the TUS coating was assessed by measuring its underwater oil-repellency from 25 to 70 °C. As shown in Fig. 7c, the coating maintained UWOCAs greater than 150° and SAs below 5° throughout the tests, demonstrating exceptional anti-oil-fouling capability at elevated temperatures. The reliable performance under thermal stress is critical for real-world applications where operational temperatures fluctuate.

The TUS coating exhibits excellent anti-fogging capability under high-humidity conditions, as evidenced in Fig. 7d. The coated area (right) retains high transparency, while the uncoated area (left) is obscured by fogging. This is because the coating's superhydrophilicity causes condensate to form a thin, continuous water film that minimizes light scattering instead of discrete droplets. The dual functionality, anti-fogging in air and anti-oil-fouling in water, renders the TUS coating highly suitable for demanding applications like underwater viewing windows, which require both optical clarity in humid air and oil repellency when submerged.

**Fig. 8:**
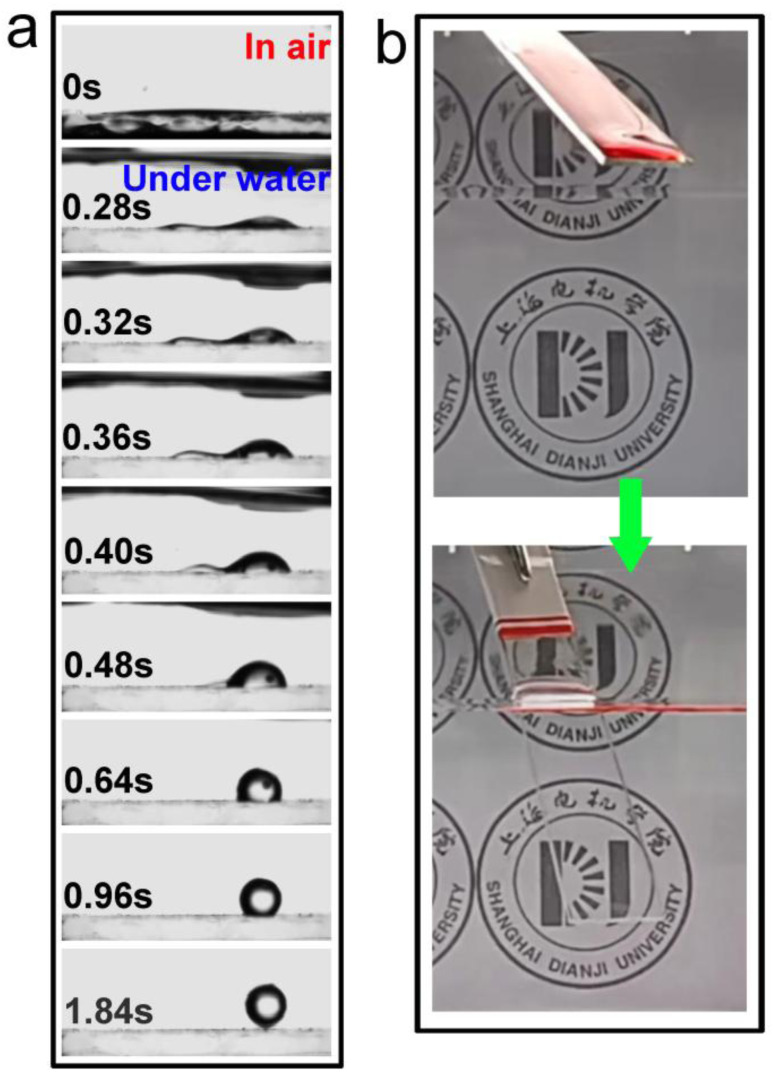
(a) Sequential images showing a mineral-oil-fouled TUS coating upon water immersion, where the oil film undergoes rapid dewetting, contracts into droplets, and detaches within 1.84 s. (b) Shedding of waste engine oil (stained with Oil Red O) from a contaminated TUS coating upon water immersion, restoring optical transparency.

The self-cleaning capability of the TUS coating, arising from its ultralow oil adhesion, was further quantified and validated through dynamic dewetting tests. As shown in Fig. 8a, a mineral oil film on the coating surface rapidly retracted and detached within 1.84 seconds upon water immersion (Movie S7). To assess practical relevance, the test was repeated with challenging waste engine oil (stained with Oil Red O, Fig. 8b). Remarkably, the TUS coating spontaneously and completely shed the contaminant, restoring its original clarity and making the underlying patterns distinctly visible. The effective removal of a complex oil underscores the coating's ability to maintain transparency in realistic, contaminated environments, attributable to the stability of its surface hydration layer.

**Fig. 9:**
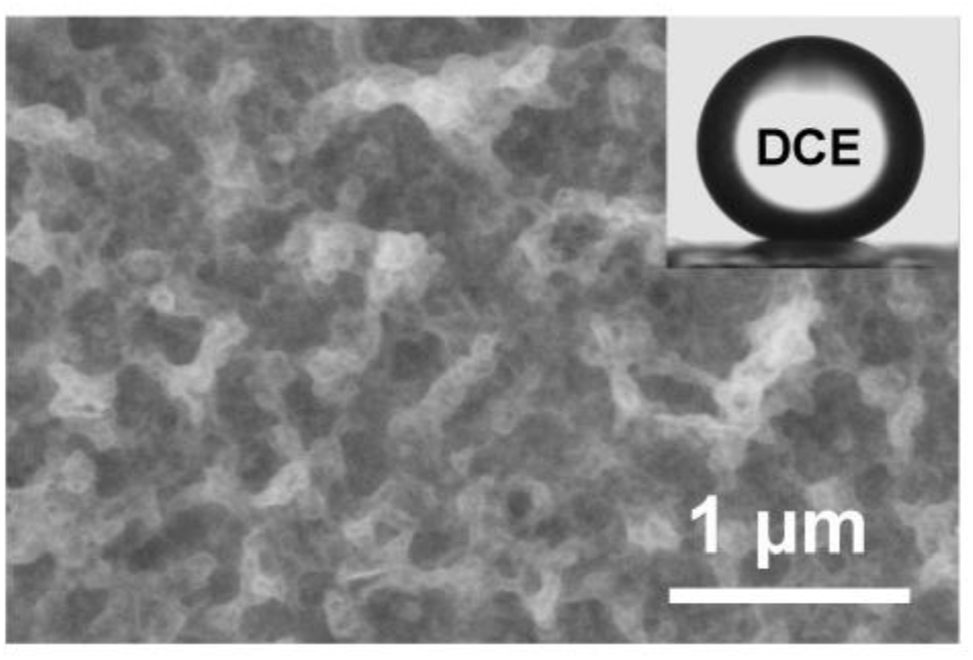
SEM image of the TUS coating after exposure to hydrodynamic shear (0.5 m s^−1^ for 12 hours), with an inset showing the retained underwater superoleophobicity (UWOCA >150° using DCE).

The TUS coating's durability against hydrodynamic shear was evaluated by exposing it to a continuous water stream (0.5 m s^−1^ for 12 hours, Fig. S8). The coating retained its structural integrity and anti-fouling performance, as confirmed by post-test SEM analysis and a maintained UWOCA >150° (DCE, Fig. 9). The result demonstrates robust mechanical stability and exceptional resistance to hydraulic shear, a critical attribute for long-term service in dynamic aquatic environments.

**Fig. 10:**
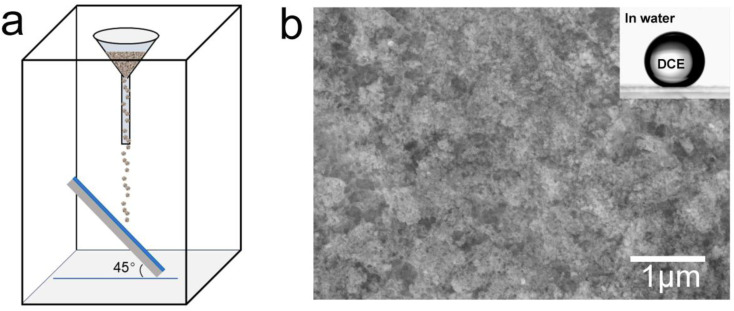
(a) Schematic of the particle impact test. (b) SEM image of the TUS coating after impact, showing surface damage. Inset: underwater oil contact angle (154° ± 3°) on the impacted coating.

To evaluate mechanical abrasion resistance, a particle impact test was performed by using titanium powder (quasi-spherical, 5–80 µm, density ∼4.5 g cm^−3^) as a model abrasive. The sample was tilted at 45° in air, and particles were dropped from a height of 15 cm (impact velocity ≈1.7 m s^−1^) at a controlled flux of 0.1 g s^−1^ for 10 s, concentrated onto a fixed spot (*Φ* ≈ 10 mm) (Fig. 10a and Movie S8). After impact, SEM revealed localized crushing and removal of nanoprotrusions (Fig. 10b). Nevertheless, the UWOCA for DCE remained above 150° (154° ± 3°), with sliding angle <5°. The TUS coating thus exhibits good mechanical robustness under concentrated abrasive stress.

### 3.6 Comparison with state-of-the-art transparent underwater superoleophobic coatings

To better contextualize the overall performance of the TUS coating, we compared its key properties, including optical transparency, underwater oil contact angle, mechanical robustness, and environmental stability, with those of recently reported transparent underwater superoleophobic coatings. Table 2 summarizes this comparison. As can be seen, the TUS coating exhibits a high underwater transparency of 92.3% and a UWOCA of 169°, which are among the highest values reported for such coatings. Importantly, in contrast to most reported transparent underwater superoleophobic coatings, our work includes systematic particle impact abrasion testing, providing direct evidence of mechanical durability alongside high salinity, thermal, and hydrodynamic stability. The combination of high transparency, robust oil repellency, and multi-scenario durability has rarely been achieved in a single coating.

**Table 2:** Comparison of key properties of recently reported transparent underwater superoleophobic coatings

Coating material	Transparency in water (T@550 nm)	UWOCA (oil type)	Mechanical test	Durability conditions	Ref.
PAA-functionalized porous silica	92.3%	169° (DCE)	Particle impact (Ti powder, 15 cm, 0.1 g s^−1^, 10 s)	20 d seawater, 70 °C, 0.5 m s^−1^ flow	This work
Nacre-inspired mineralized film	∼88%	∼162° (DCE)	Sand impingement	30 d seawater	2
PNIPAM-clay nanocomposite hydrogel	∼88%	159° (DCE)	Compression	—	12
Zwitterionic polymer coating	>90% (at 650 nm)	∼165° (hexadecane)	Hydrodynamic shear (0.3 m s^−1^, 24 h)	pH 2–12, seawater, surfactant	3
PVA/silica hybrid (immersion-cured)	∼65%	∼160° (crude oil)	Sand impact	5 d seawater, acid, alkali	27
Chitosan/CaCO_3_ nacre-like film	>80%	151.2° ± 1.8° (DCE)	—	—	29
Triple-defense coating (PDA/PEI-g^+^/PAA/ACC)	∼93% (wet)	>155° (crude oil)	Sandpaper abrasion, tape peeling	Seawater, pH 4–12	28

## 4 Conclusion

In conclusion, we have developed a transparent underwater superoleophobic (TUS) coating that simultaneously exhibits high optical transparency (92.3% in water at 550 nm), good oil repellency (UWOCA up to 169°), and moderate resistance to mechanical abrasion, prolonged seawater exposure, elevated temperature, and hydrodynamic shear. The TUS coating offers a compelling balance of transparency, oil repellency, and environmental durability for applications with moderate wear conditions. Such performance was achieved by covalently anchoring high-molecular-weight PAA chains onto a mechanically stable, porous nano-silica scaffold. The resulting hybrid coating combines the mechanical stability of the inorganic framework with the hydration capability of the polymer, yielding an underwater oil contact angle of 169° ± 2°, a sliding angle of 1.5° ± 0.3°, and an ultralow adhesion force of ≈0.48 µN. The TUS coating also exhibits anti-fogging performance and self-cleaning ability against complex oils such as waste engine oil. Notably, the TUS coating maintains its anti-oil-fouling performance and optical clarity under a range of realistic stressors, including high salinity (3.5 wt% NaCl, 20 days), elevated temperature (up to 70 °C), hydrodynamic shear (0.5 m s^−1^, 12 h), and particle impact abrasion. This work therefore offers a feasible strategy for designing durable, transparent, and oil-repellent coatings for underwater optical devices and marine equipment.

## Author contributions

L. Xu: conceptualization, supervision, project administration, writing – review and editing. J. Li: investigation, validation, formal analysis, writing – original draft. R. Li: investigation, resources, methodology, data curation. X. Lu: software, formal analysis. M. Yang: investigation, data curation, visualization. P. Gao: investigation, validation. L. Tong and S. Zhao: writing – review and editing, supervision. H. Jiang: software.

## Conflicts of interest

There are no conflicts to declare.

## Supplementary Material

RA-016-D6RA05313K-s001

RA-016-D6RA05313K-s002

RA-016-D6RA05313K-s003

RA-016-D6RA05313K-s004

RA-016-D6RA05313K-s005

RA-016-D6RA05313K-s006

RA-016-D6RA05313K-s007

RA-016-D6RA05313K-s008

RA-016-D6RA05313K-s009

## Data Availability

The data supporting this article have been included as part of the supplementary information (SI). Supplementary information is available. See DOI: https://doi.org/10.1039/d6ra05313k.
