# Supplementary material for: Highly transparent PAA-functionalized porous silica coatings with underwater superoleophobicity and oil-fouling resistance
Source: RSC Adv. 2026 Jul 13;16(37):40177–88. doi: 10.1039/d6ra05313k (PMC13361208; doi:10.1039/d6ra05313k)
Supplement: Supplementary file 1 [file RA-016-D6RA05313K-s009.pdf]

Supplementary Information (SI) for RSC Advances.
This journal is © The Royal Society of Chemistry 2026

Supplementary Information

Highly Transparent PAA-Functionalized Porous Silica Coatings with Underwater Superoleophobicity and Oil-Fouling Resistance

*Lianyi Xu**[a], *Jinlong Li*[a], *Rongbin Li*[a], *Xuemin Lu*[b], *Miaosen Yang*[a], *Pan Gao*[a], *Liping Tong*[a], *Su Zhao*[a], *Haiyang Jiang*[a]

a School of Materials Science and Engineering, Shanghai Dianji University, Shanghai, 201306, P.R. China.

*E-mail: xuly@sdju.edu.cn

b School of Chemistry and Chemical Engineering, State Key Laboratory of Metal Matrix Composite, Shanghai Jiao Tong University, 800 Dongchuan Road, Shanghai, 200240, China

S1. Preparation of highly transparent and mechanically stable porous nano-silica (TMNS) coatings

As described in our previous work (*J. Mater. Chem. A*, 2015, 3, 3801), highly transparent and mechanically robust nanoporous silica (TMNS) coatings were fabricated on ITO glass in three steps [32]. In this work, all electrochemical experiments were performed in a standard three-electrode cell at room temperature and under relative humidity below 40%. The applied potential was controlled by a CHI630E electrochemical analyzer. The working electrode was indium tin oxide (ITO) glass (< 10 Ω sq^{-1}), which was ultrasonically cleaned sequentially in deionized water and anhydrous ethanol for 1 minute each, followed by drying under a stream of N_2. A platinum wire (1 mm diameter) served as the counter electrode and was cleaned prior to each experiment. An Ag/AgCl wire was used as a quasi-reference electrode.

First, transparent, porous network-like PEDOT templates were electrodeposited onto the ITO substrate via cyclic voltammetry (CV) from an acetonitrile solution containing 0.01 M EDOT monomer and 0.2 M $LiClO_4$ as the supporting electrolyte (Figure S1). The amount of PEDOT deposited was controlled by varying the electrodeposition charge density between 10 and 60 mC cm^{-2}. Doping and dedoping of the films were carried out by potentiostatic methods in a monomer-free 0.2 M $LiClO_4$/acetonitrile electrolyte. After deposition, all films were rinsed twice with acetonitrile (approximately 1 mL each) and dried under a N_2 stream at room temperature before characterization. The SEM image of the as-prepared PEDOT films, which reveals a porous network-like nanostructure, is presented in Figure S2.

Subsequently, the as-prepared PEDOT films were placed in a sealed desiccator, and tetraethyl orthosilicate (TEOS) was deposited via ammonia-catalyzed chemical vapor deposition (CVD) for 48 hours. Finally, the PEDOT-silica hybrid coatings were calcined at 530 °C for 2 hours in air to remove the PEDOT template, resulting in highly transparent and mechanically robust nanoporous silica (TMNS) coatings that replicated the morphology of the original PEDOT films. As verified in our previous study, the resulting TMNS coatings exhibited robust mechanical stability, attributed to covalent linkages between the silica nanostructure and the ITO substrate.

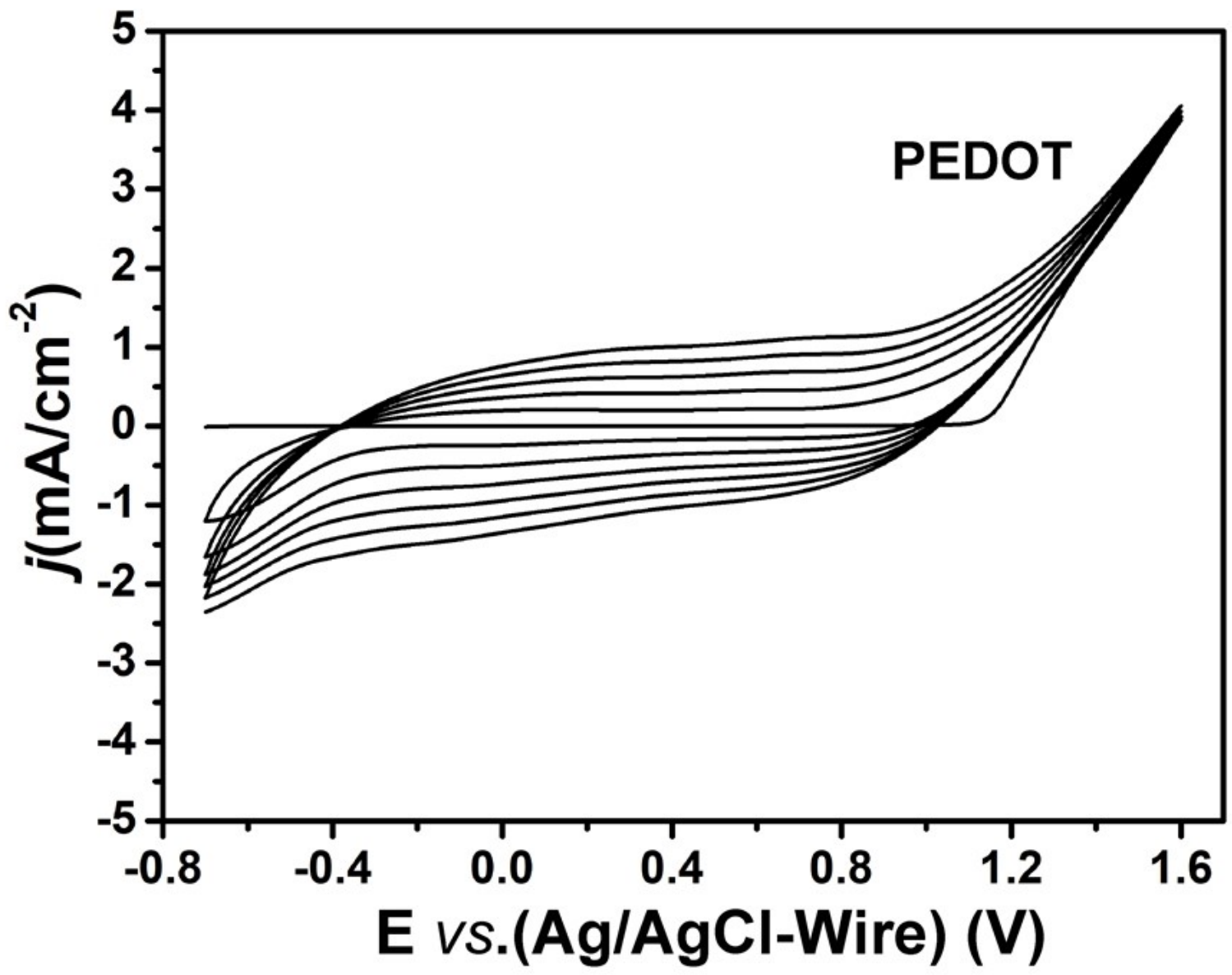

Figure S1. Electrochemical polymerization of the PEDOT template. Consecutive cyclic voltammograms (from -0.7 V to 1.6 V vs. Ag/AgCl, scan rate: 200 mV s^{-1}) recorded during the electrodeposition of PEDOT from an acetonitrile solution containing 0.01 M EDOT and 0.2 M $LiClO_4$. The progressive increase in current density with cycle number indicates the continuous growth of the conductive polymer network on the ITO substrate. The mass of deposited PEDOT was precisely controlled by terminating the process at a charge density between 10 and 60 mC cm^{-2}.

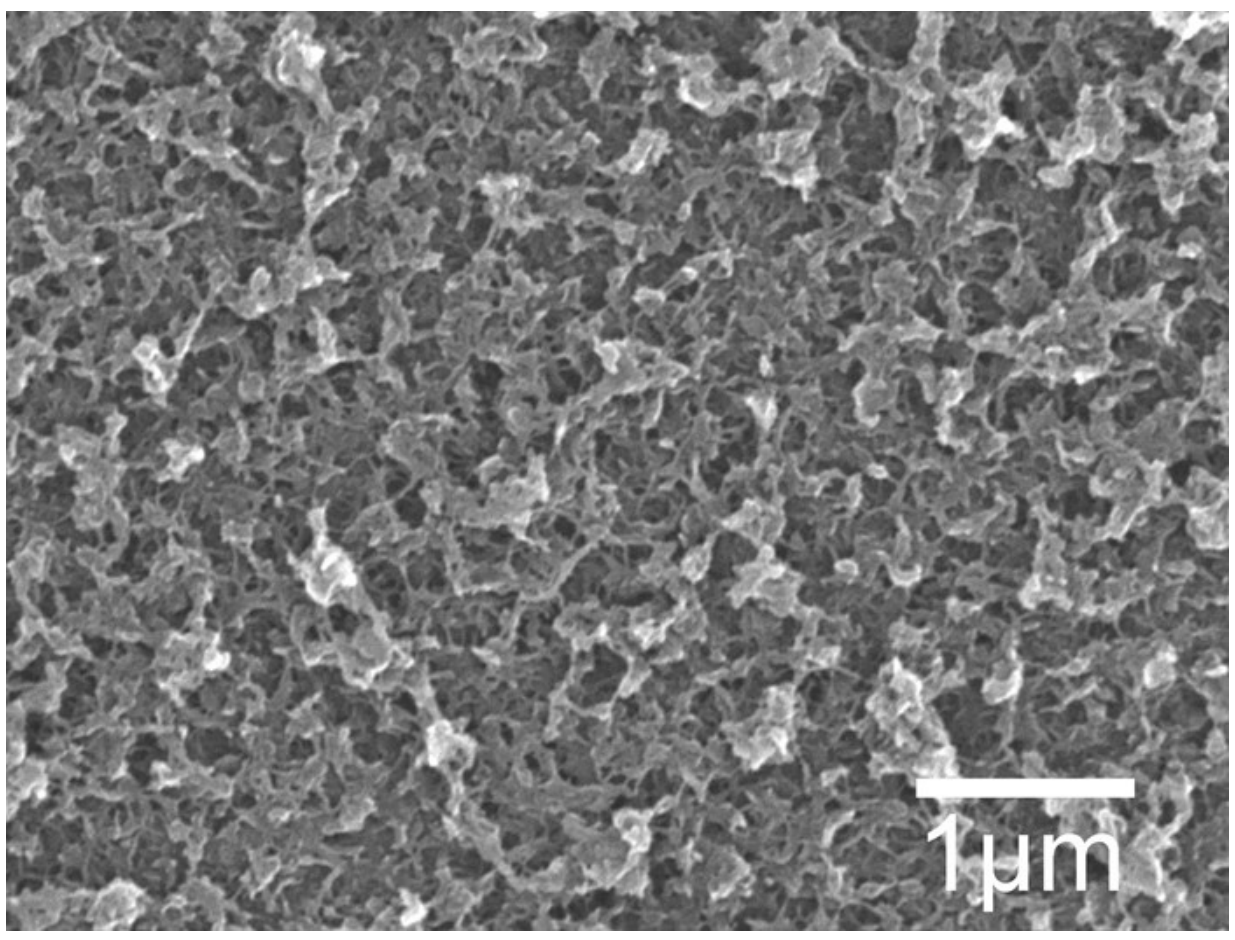

Figure S2. Morphology of the as-prepared PEDOT template. Representative SEM image of the electrodeposited PEDOT film, confirming the formation of a continuous, porous, and network-like nanostructure. The porous network serves as the template for the subsequent CVD of silica precursors.

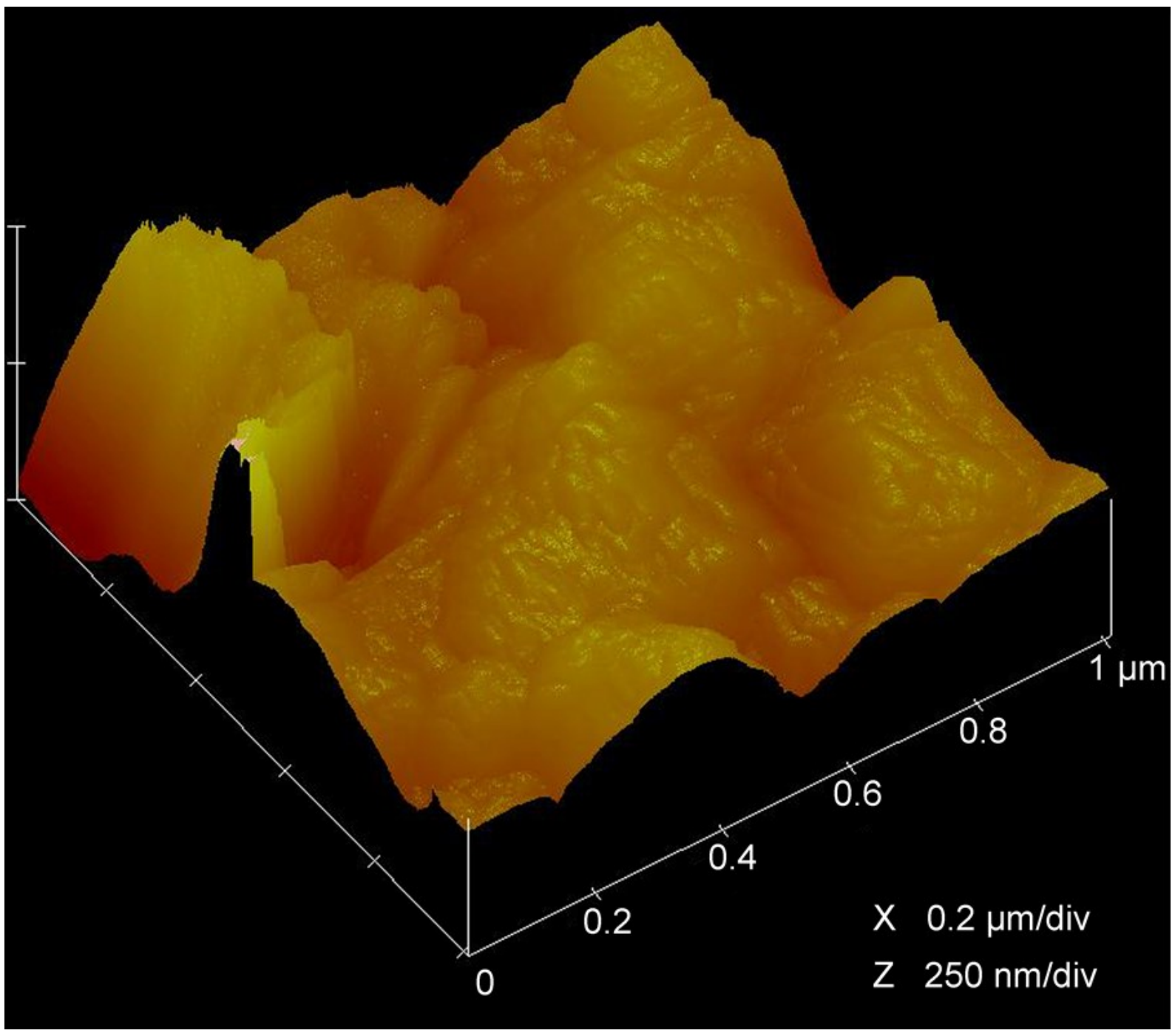

Figure S3. AFM topography image of the TUS coating, demonstrating the preserved nanoprotrusive surface.

S2. FT-IR Spectroscopy Confirms PAA Grafting via Amide Bond Formation

FT-IR spectroscopy confirmed the successful grafting of PAA onto the underlying TMNS coating via amide bond formation. As shown in Figure S4, the characteristic C=O stretching vibration of pure PAA (carboxylic acid at 1711 cm^{-1}, see blue line) shifts to 1664 cm^{-1} (amide I band) in the TUS coating (see red line). Concurrently, a new amide II band emerges at 1554 cm^{-1} (see red line). This spectral evolution is consistent with the reference product from the reaction between PAA and APTES (see black line), conclusively indicating the conversion of carboxylic acids to amide linkages.

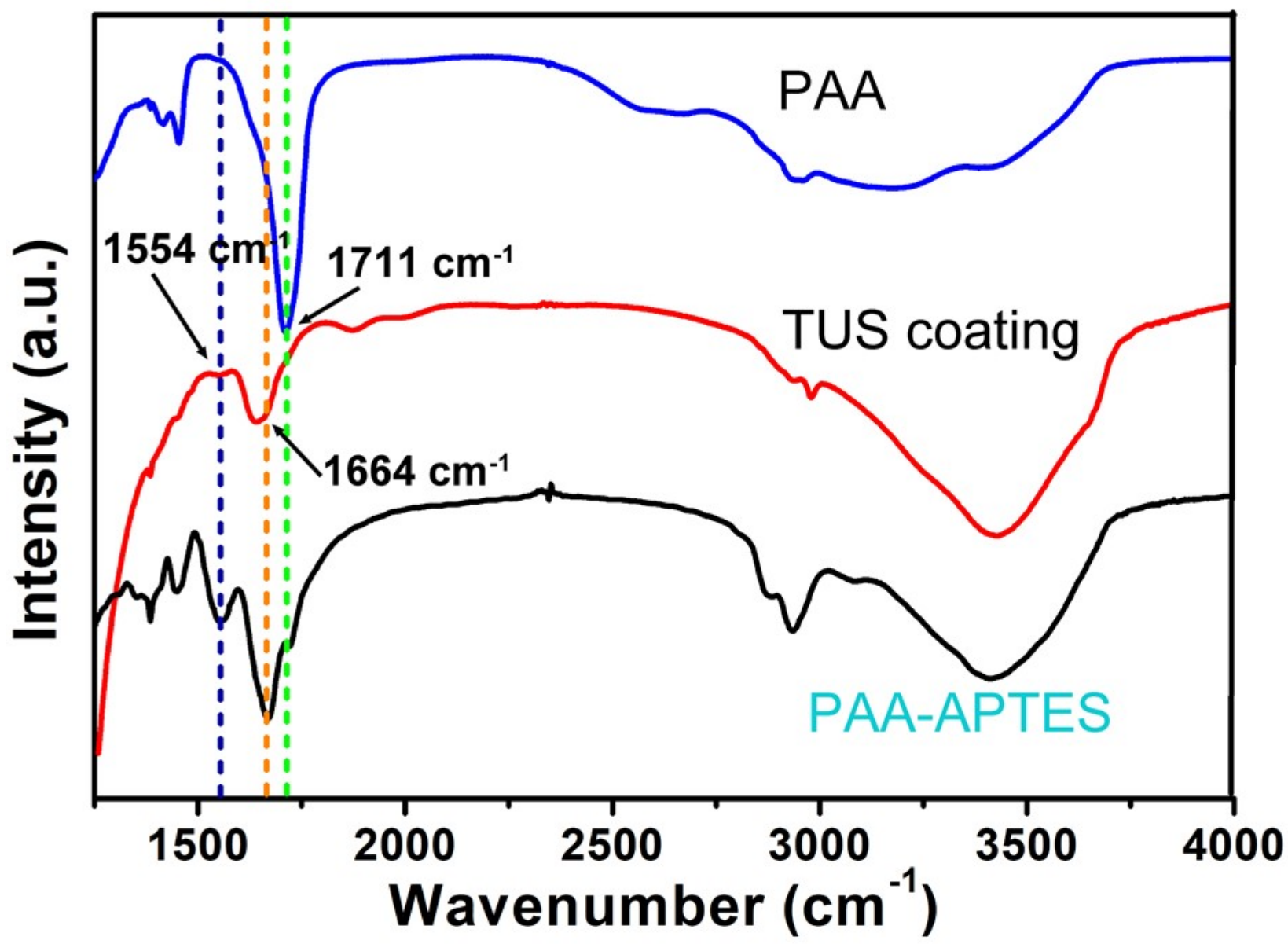

Figure S4. FT-IR spectra confirming amide bond formation. The spectrum of the final TUS coating (red line) shows a redshifted C=O stretch (Amide I band) at 1664 cm⁻¹ and a new Amide II band at 1554 cm⁻¹ compared to pure PAA (blue line, 1711 cm⁻¹). These features, which match the PAA-APTES reference product (black line), provide direct evidence for the covalent grafting of PAA onto the TMNS coating.

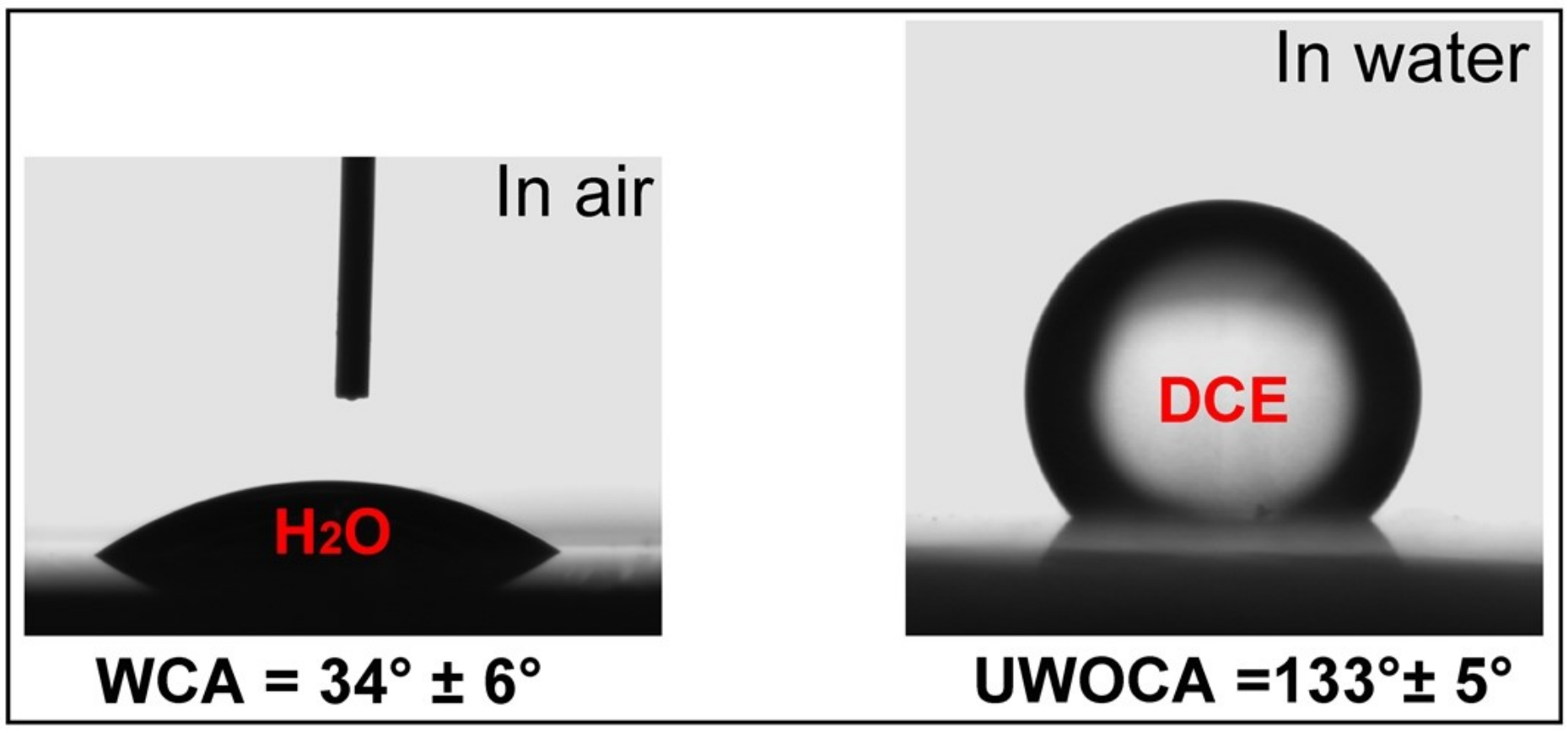

Figure S5. The contact angle measurements on a flat glass surface. The left and right images display the water contact angle in air (WCA = 34° ± 6°) and the underwater oil contact angle (UWOCA = 133° ± 5°), respectively.

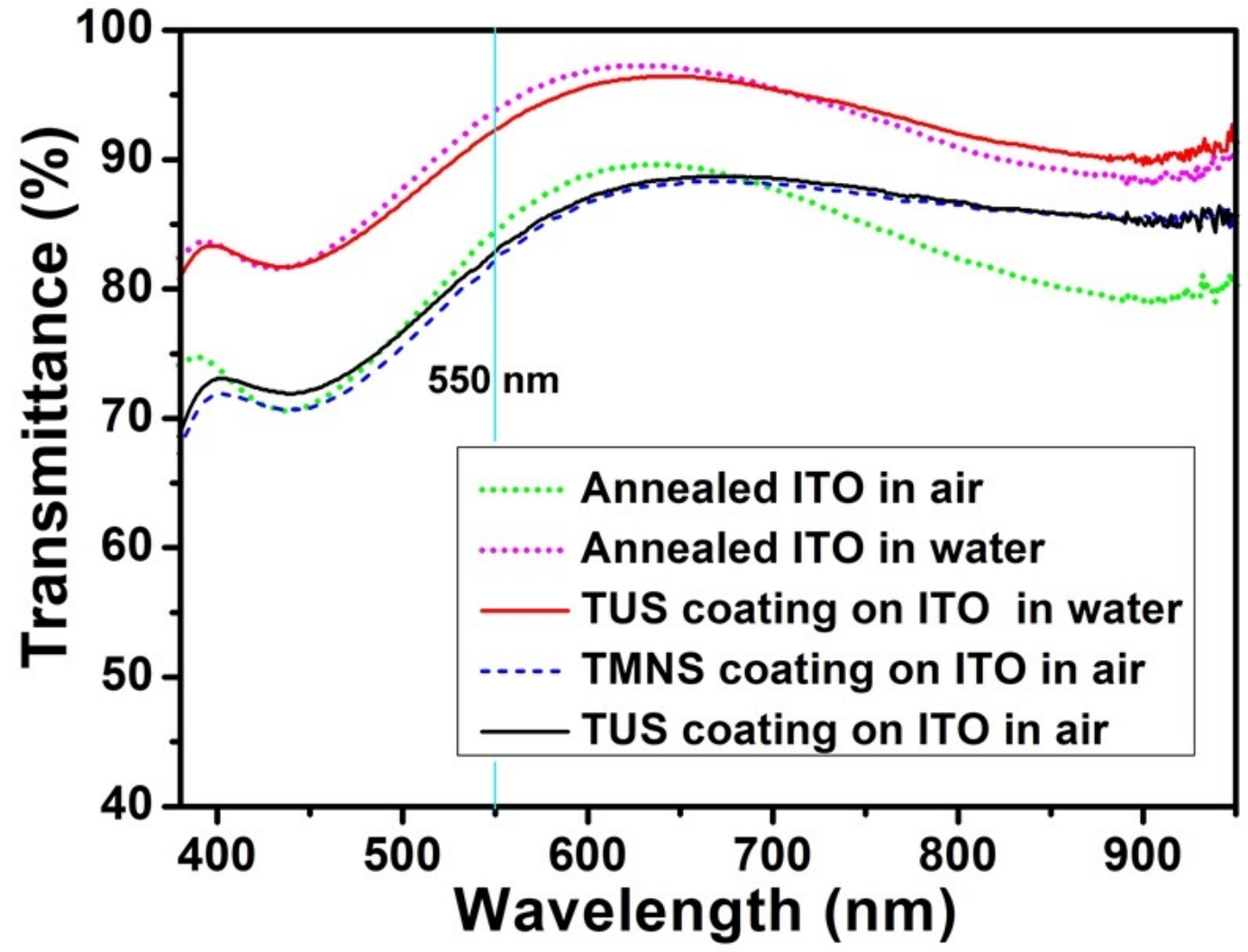

Figure S6. Comparison of the optical transmittance of TUS coating and bare annealed ITO glass measured in air and in water at a wavelength of 550 nm. The TUS coating exhibits a transmittance gain of 9.4% (from 82.9% to 92.3% at 550 nm) and the ITO glass shows a transmittance gain of 9.4% (from 84.4% to 93.8% at 550 nm).

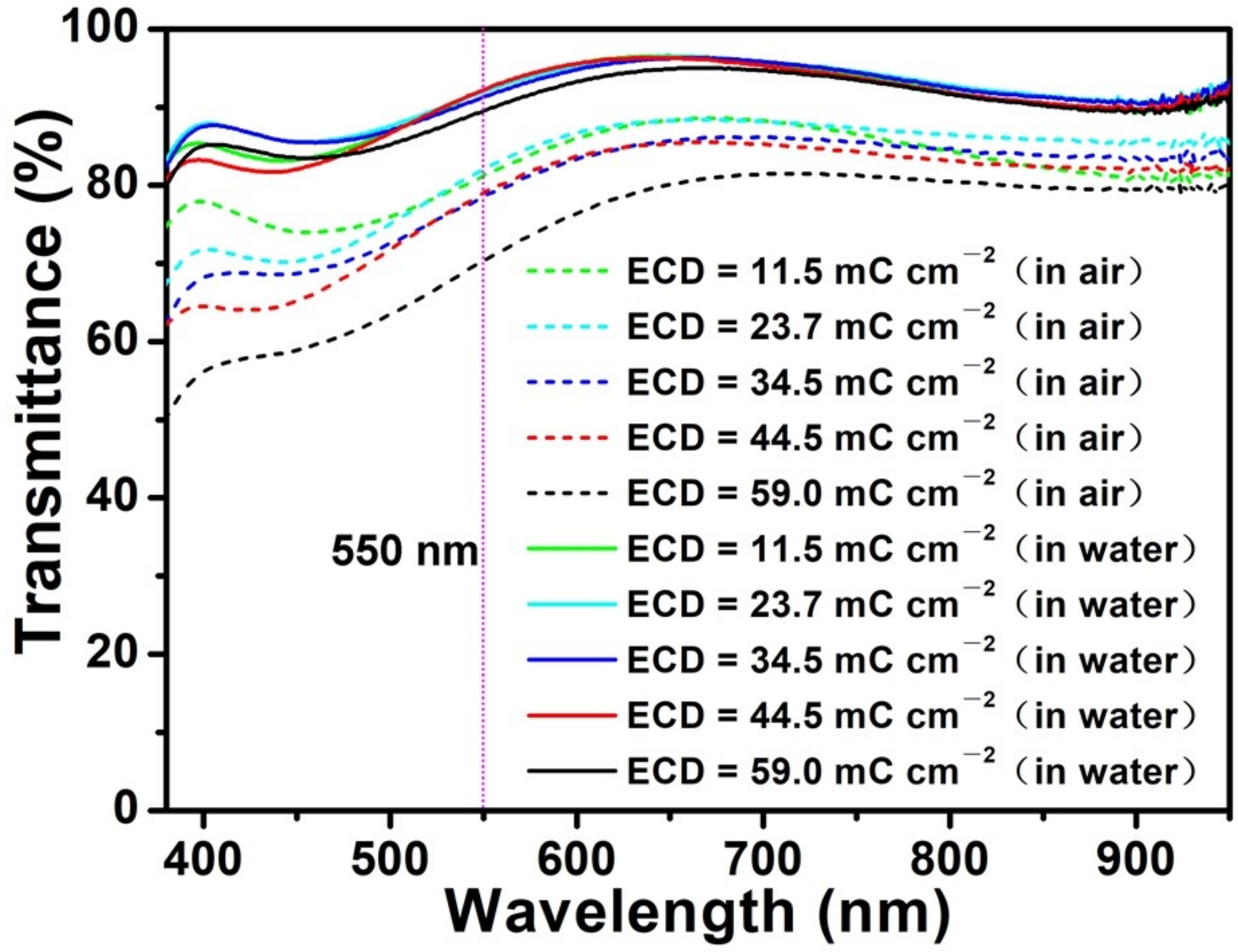

Figure S7. Transmittance spectra of TUS coatings fabricated with varying PEDOT electrodeposition charge densities (ECD, ranging from 11.5 to 59 mC cm^{-2}), measured in both air and water. In air (dashed lines), transmittance (at 550 nm) decreases as the charge density increases, which is attributed to enhanced light scattering from greater surface roughness. In water (solid lines), all coatings exhibit consistently high transmittance with minimal variation, confirming that water

penetration minimizes refractive index contrasts and effectively suppresses scattering, ensuring robust underwater transparency independent of the fabrication charge density.

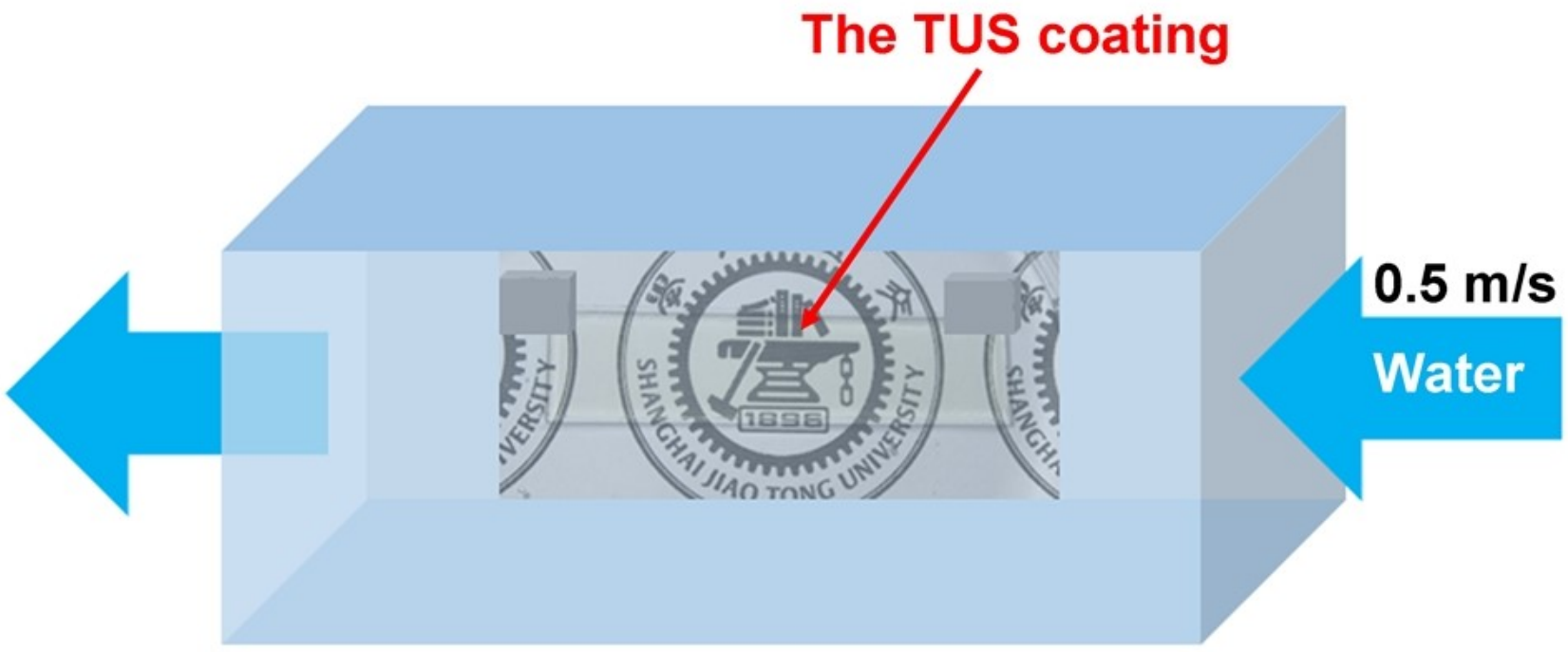

Figure S8. Schematic of the experimental setup for evaluating the mechanical durability of the TUS coating under a constant water flow of 0.5 m/s for 12 hours.

Movie S1. Real-time spreading of a 4 μL water droplet on the TUS surface in air, showing complete wetting within 2.0 s.

Movie S2. A water droplet rapidly rolls off the TUS surface upon minimal tilting.

Movie S3. A DCE oil droplet is cyclically compressed and completely detaches from the submerged TUS coating without any residue.

Movie S4. A DEC oil droplet sliding on the TUS coating, demonstrating its ultralow contact angle hysteresis.

Movie S5. Strong adhesion of a DCE oil droplet to the TMNS coating during the compression-detachment test.

Movie S6. Dynamic process of a water droplet being absorbed and spreading on the TUS coating under hexadecane, leading to complete wetting within 26 seconds.

Movie S7. Dynamic demonstration of mineral oil film retracting and detaching from the surface in water within 1.84 seconds.

Movie S8. Particle impact test on the TUS coating (Ti powder, 15 cm height, 10 s).
